# Antiparasitic activity of Colombian Amazon palm extracts against *Giardia lamblia* trophozoites: insights into cellular death mechanisms

**DOI:** 10.3389/fmicb.2025.1523880

**Published:** 2025-03-19

**Authors:** Juan Javier García-Bustos, Gabriel Luna Pizarro, Rocío G. Patolsky, Mariana Belén Joray, Vivian Villalba-Vizcaino, Paula Galeano, Fabián Espitia-Almeida, Marco Correa Múnera, Mehmet Ozturk, Andrea S. Rópolo, Constanza Feliziani, María Carolina Touz, Jerónimo Laiolo

**Affiliations:** ^1^Programa de Medicina Veterinaria y Zootecnia, Universidad de La Amazonia, Caquetá, Florencia, Colombia; ^2^Universidad del Magdalena, Facultad Ciencias de la Salud, Doctorado en Medicina Tropical SUE-Caribe, Grupo de Investigación en Inmunología y Patologia (GIPAT), Santa Marta, Colombia; ^3^Instituto de Investigación Médica Mercedes y Martín Ferreyra, Consejo Nacional de Investigaciones Científicas y Técnicas (INIMEC-CONICET), Universidad Nacional de Córdoba, Córdoba, Argentina; ^4^Centro de Investigación y Desarrollo en Inmunología y Enfermedades Infecciosas, Consejo Nacional de Investigaciones Científicas y Técnicas (CIDIE-CONICET-UCC), Universidad Católica de Córdoba, Córdoba, Argentina; ^5^Facultad de Ciencias Básicas, Universidad de La Amazonia, Caquetá, Florencia, Colombia; ^6^Centro de Investigaciones en Ciencias de la Vida, Facultad de Ciencias Básicas y Biomédicas, Universidad Simón Bolívar, Barranquilla, Colombia; ^7^Facultad de Ciencias Básicas, Programa de Biología, Universidad del Atlántico, Puerto Colombia, Colombia; ^8^Department of Chemistry, Faculty of Science, Mugla Sitki Koçman University, Mugla, Türkiye; ^9^Faculty of Chemistry and Chemical Technology, Al-Farabi Kazakh National University, Almaty, Kazakhstan; ^10^Facultad de Ciencias de la Salud, Universidad Católica de Córdoba, Córdoba, Argentina

**Keywords:** Colombian Amazon, *Giardia lamblia*, giardicidal activity, medicinal plants, plant extracts

## Abstract

**Introduction:**

Colombian plants have a long history of use in traditional medicine and ethnopharmacology, particularly for treating stomach pain, digestive issues, diarrhea, and other gastrointestinal disorders. Recent studies have renewed interest in their potential therapeutic properties.

**Methods:**

This study evaluated the giardicidal activity of 15 crude plant extracts native to the Colombian Amazon against Giardia lamblia (genotype A, strain WB/1267). The MTT colorimetric assay was used to determine the effectiveness of these extracts at a concentration of 500 μg/mL. Extracts showing significant activity were further analyzed to determine their half-maximal inhibitory concentration (IC50). The cell death mechanisms of Attalea butyracea were studied using flow cytometry, confocal microscopy, and transmission electron microscopy (TEM).

**Results:**

Among the tested extracts, the *Attalea butyracea* fruit extract (**P-2**) exhibited the highest activity against WB/1267 (IC_50_ = 62.10 ± 6.57 μg/mL) and demonstrated giardicidal activity against GS/M (IC_50_ = 100.90 ± 3.40 μg/mL, genotype B) human infecting strains. These results prompted a detailed investigation into its mechanism of action using the WB/1267 strain as a model. At its IC_50_ concentration, **P-2** primarily exerted its antiproliferative effect by induction of early apoptosis. A notable increase in late apoptosis and necrosis was observed at 2xIC_50_. Immunofluorescence assay (IFA) and confocal microscopy revealed chromatin condensation in treated trophozoites, while flow cytometry indicated G1/S cell cycle arrest. Furthermore, exposure to **P-2** led to oxidative stress, evidenced by a significant increase in reactive oxygen species (ROS). The extract’s ability to disrupt various structural components of the parasite was confirmed through IFA and transmission electron microscopy. Interestingly, the **P-2** extract effectively synergized with the first-line drug metronidazole against *Giardia* WB/1267 trophozoites.

**Discussion:**

These findings underscore the therapeutic potential of Colombian plant extracts in treating giardiasis, particularly highlighting the novel giardicidal activity of *Attalea butyracea* fruit extract and its promise for further therapeutic development.

## 1 Introduction

*Giardia lamblia* (syn: *G. intestinalis*, *G. duodenalis*) is a parasitic protozoan that lives in the small intestine of humans and other vertebrates. This parasite causes giardiasis, a disease with symptoms that can vary from asymptomatic cases to diarrhea, steatorrhea, abdominal cramps, bloating, nausea, malabsorption, and, in some instances, progressing to chronic conditions (Leung et al., 2019; Buret et al., 2020). These conditions not only predispose patients to anemia, malnutrition, growth stunting, and cognitive delays but also increase the risk of post-infectious complications such as irritable bowel syndrome and chronic fatigue (Robertson et al., 2010; Fink and Singer, 2017). *G. lamblia* is globally recognized as the third most prevalent cause of diarrheal disease among children under 5 years of age, with an estimated annual incidence of giardiasis-associated diarrhea exceeding 200 million cases across all age groups (Cernikova et al., 2018; Fakhri et al., 2021). This protozoan is commonly found in water, food, and surfaces contaminated with feces from infected animals or humans (Leung et al., 2019). The infection is transmitted through the fecal-oral route, so poor personal hygiene and sanitation are important transmission risk factors (Ryan et al., 2019). The life cycle of *G. lamblia* is simple. It comprises two well-differentiated stages: the trophozoite, the infective form that colonizes the small intestine, and the cysts, resistant forms, that cause disease transmission. Infectious cysts can survive in the environment for several months, contributing to the strong association between infection and contaminated water or food. The trophozoite is pear-shaped and characterized by two transcriptionally active nuclei, a cytoskeleton, and peripheral vacuoles (PVs) that function as endosomes and lysosomes. Additionally, it adheres to the epithelium of the small intestine using a ventral adhesive disk. Furthermore, it possesses four pairs of flagella for mobility and organelles analogous to mitochondria known as mitosomes (Feliziani et al., 2015; Hagen et al., 2020; Lagunas-Rangel et al., 2021). Of the eight described genotypes (Assemblages) of *Giardia* (A-H), only two infect humans: genotypes A and B, which also have zoonotic potential (Binz et al., 1992; Thompson and Lymbery, 1996).

Current therapy for giardiasis relies on treatment with various classes of antimicrobial drugs available (Mørch and Hanevik, 2020). The most widely used worldwide are members of the 5-nitroimidazole family, such as metronidazole (MTZ) and tinidazole (Gardner and Hill, 2001; Loderstädt and Frickmann, 2021). However, this first-line therapy fails in up to 20% of cases and may contribute to the development of resistance to giardicidal drugs. Different resistance mechanisms have been observed for MTZ, benzimidazoles such as albendazole (ABZ) and mebendazole, as well as cross-resistance between different drugs, such as MTZ co-resistance with nitazoxanide (Ansell et al., 2015; Emery-Corbin et al., 2021). Managing such protozoal infections involves systemic treatments that often demand prolonged medication use and are associated with notable side effects. Furthermore, this first-line therapy is ineffective against resistant strains (Leung et al., 2019). Notably, over the past 25 years, no randomized controlled trials have been specifically designed to study or evaluate the treatment of infections that are resistant to standard treatments (refractory infections) (Mørch and Hanevik, 2020). Hence, the necessity arises to identify new chemical entities capable of reaching the target cell without affecting the rest of the organism. Only a handful of drugs are currently in use to treat infections caused by pathogenic protozoan parasites, and in many instances, without medical treatment, the infection can be fatal (Supuran, 2023).

The utilization of plants for medicinal purposes can be traced back to prehistoric eras, supported by evidence from archeological discoveries (Hardy et al., 2012). Throughout human history, various cultures have empirically utilized plants for their therapeutic properties, often through oral consumption or the preparation of crude extracts (Chaachouay and Zidane, 2024). Ethnopharmacology has played a crucial role in uncovering the therapeutic potential of plant-based remedies. By studying indigenous knowledge and practices, ethnopharmacologists have identified numerous bioactive compounds with medicinal properties, contributing to the development of modern pharmaceuticals (Pirintsos et al., 2022).

Colombia is renowned for its exceptional biodiversity, encompassing approximately 10% of the world’s total (Gori et al., 2022). The Amazon region alone is estimated to host a significant portion of Colombia’s biodiversity, with an estimated 9,055 plant species representing a rich reservoir of potential medicinal resources (Ministerio de Ambiente y Desarrollo Sostenible, 2022). However, despite this vast diversity, only 119 plant species are currently cataloged in the Colombian Vademecum of Medicinal Plants (Ministerio de la Protección Social, 2008). Medicinal plants utilized in the treatment of gastrointestinal disorders and ethnodiseases such as stomach pain, digestive issues, dysentery, and diarrhea could serve as an important source of novel antiprotozoal drugs with high efficacy and safety (Calzada and Bautista, 2020; Ranasinghe et al., 2023). Exploring these plants further may unlock valuable therapeutic compounds to address the growing challenge of drug-resistant protozoal infections. These plants have been traditionally utilized in ethnopharmacology for their therapeutic properties against various ailments, especially gastrointestinal disorders.

This study aimed to thoroughly assess the giardicidal activity of 15 crude extracts from the diverse flora of the Colombian Amazon. Among the tested extracts, the fruit extract of *Attalea butyracea*, commonly known as “Palma de vino” (wine palm), demonstrated the highest activity against *Giardia lamblia* WB/1267 (genotype A) trophozoites. Further analysis revealed that the extract caused significant cell damage, likely through an apoptotic mechanism of parasite death. The extract’s efficacy was then evaluated against *G. lamblia* GS/M (genotype B) trophozoites to assess whether it also induces cell damage in the other human-infective genotype. These findings expand the pharmacological understanding of Amazonian botanicals and may provide valuable insights for developing natural therapies against giardiasis, particularly in cases where conventional treatments are ineffective.

## 2 Materials and methods

### 2.1 Plant collection and extract preparation

The plants were collected in the Uitoto Huitorá Indigenous Reserve, situated in the northern region of the Amazon River basin, Caquetá department, Colombia (between 0°9′41.064″ N latitude and 74°40′28.282″ W longitude) in March 2022. Voucher specimens (Supplementary Table S1) have been deposited in the “Enrique Forero – HUAZ” Herbarium of the Amazonia University and were authenticated by botanist Marco Correa. Each plant’s air-dried and powdered aerial parts were extracted through 72-h maceration in a hydroethanolic solution of 70% ethanol and 30% water, with a plant-to-solvent ratio of 1:10 (w/v). The yields of each extract, after solvent removal, expressed as a percentage of the weight of the crushed plant material, are presented in Supplementary Table S1. The high-performance liquid chromatography (HPLC) profile of *Attalea butyracea* fruit extract was performed on a Shimadzu LC-20 AT CE (Shimadzu Corp., Tokyo, Japan), equipped with a Shimadzu SPD-M20A detector using a Phenomenex Prodigy 5 *μ* ODS (4.6 mm i.d. X 250 mm) reversed-phase column. The mobile phase was 90% acetonitrile in water, with UV detection at 190 nm.

### 2.2 *Giardia lamblia* culture

*G. lamblia* trophozoites isolates WB clone 1267 (ATCC 50582, Genotype A) and GS/M (ATCC 50581, Genotype BIV) were purchased at American Type Culture Collection.^1^ The trophozoites were cultured axenically in TYI-S-33 medium (pH 7), supplemented with 10% adult bovine serum and 0.05% bovine bile (complete growth medium). Cultures were grown in borosilicate tubes with 14 mL of medium and 100 μL of trophozoites. Incubation was carried out at 37°C with a 45° angle. After 1 h, trophozoite adherence was verified. Once a monolayer formed, trophozoites were harvested by incubating the tubes at 4°C for 30 min and centrifuging at 2,500 rpm for 10 min to recover them in the pellet.

### 2.3 MTT assay

To assess the cytotoxic potential of the extracts, we conducted the MTT colorimetric assay as described in a previous study (Barzola et al., 2024). Briefly, *G. lamblia* WB/1267 trophozoites at a density of 5 × 10^5^ cells per well, suspended in 150 μL of complete growth medium, were seeded into 96-well plates with an additional 150 μL of medium containing each tested extract previously dissolved in DMSO (final concentration 0.5% v/v, as this concentration showed no adverse effects on cell growth). The extracts were evaluated at a final concentration of 500 μg/mL. Promising extracts identified from the initial screening were further assessed at serial dilutions ranging from 500 to 3.91 μg/mL. Following anaerobic incubation for 4 h at 37°C, the plates were centrifuged at 2,000 rpm for 10 min. Subsequently, three washes were performed by centrifugation, and 20 μL of a 5 mg/mL MTT solution in sterile PBS was added to each well, followed by further incubation for 4 h. After removing the supernatants, 100 μL of DMSO was added to solubilize the purple formazan crystals produced by metabolically viable cells. Absorbance was measured at 570 nm using a Model 680 microplate reader (Bio-Rad, United States). Cytotoxicity percentages were determined relative to DMSO-treated control cells, considered 100% viable as they exhibited behavior similar to untreated cells. The percentage of cytotoxic activity was calculated using the formula: cytotoxicity (%) = [1 − (OD of treated cells – OD of DMSO) / (OD of control cells – OD of DMSO)] × 100, where OD refers to optical density. Half-maximal inhibitory concentrations (IC_50_) were determined from the mean values obtained from replicate wells, representing the concentrations required to inhibit 50% cell proliferation. The IC_50_ of MTZ (Sigma-Aldrich, St Louis, MO) dissolved in DMSO was also determined in the four strains as a positive control for comparison with the extract’s activity. The most active crude extract (*Attalea butyracea* fruit extract, IC_50_ = 62.10 ± 6.57 μg/mL) was evaluated for cytotoxicity against the *G. lamblia* GS/M trophozoites using the same method.

### 2.4 Annexin V/PI double staining

Flow cytometry was used to investigate the potential of *Attalea butyracea* fruit extract to induce early cellular events indicative of trophozoite cell death. Annexin V served as a marker of apoptosis and propidium iodide (PI) as an indicator of membrane damage, potentially associated with late apoptosis or necrosis. Apoptotic cells exhibit distinct morphological and biochemical characteristics. A commercially available Dead Cell Apoptosis Kit (Thermo-Fisher Scientific, United States) containing Annexin V-Alexa Fluor^®^ 488 and PI was employed, following a previously validated protocol (Barzola et al., 2024). Briefly, the trophozoites WB/1267 (5 × 10^4^ cells) were exposed to the IC_50_ and 2 × IC_50_ of the crude extract. Control groups included untreated cells as negative controls (DMSO 0.5%). After anaerobic incubation for 24 h at 37°C trophozoites were washed with cold PBS and suspended in 100 μL of 1X binding buffer. They were subsequently incubated with Annexin V-Alexa 488 and PI in the dark at room temperature for 15 min. Data was acquired using a FACSCanto II flow cytometer (Becton & Dickinson, New Jersey, NY). Analysis of Annexin V/PI dot plots, divided into quadrants, identified viable cells (Annexin V−/PI−), early apoptotic cells (Annexin V+/PI−), late or secondary apoptotic cells (Annexin V+/PI+), and necrotic cells (Annexin V−/PI+).

### 2.5 Morphological examination using DAPI fluorescence

*G. lamblia* WB/1267 trophozoites (5 × 10^5^/well) were cultured in 96-well plates and treated with **P-2** extract at IC_50_ and 2 × IC_50_ concentrations for 48 h. The resulting pellet was resuspended and fixed in 4% formaldehyde solution in 1X PBS for 40 min at room temperature. For nucleus staining, fixed cells were washed with PBS and then incubated with 1 μg/mL DAPI for 5 min protected from the light. Samples were mounted using FluorSave mounting medium (Merck Group, Darmstadt, Germany) and visualized with an Olympus FV1200 confocal microscope.

### 2.6 Cellular DNA flow cytometric analysis

WB/1267 trophozoites were harvested and fixed at 4°C overnight with 70% ethanol. The fixed cells were incubated with RNAse A (2 μg/mL) and PI (50 μg/mL) at 4°C overnight (Barzola et al., 2024). After washing, they were analyzed by flow cytometry (FACSCanto II flow cytometer, Becton Dickinson, New Jersey, NY). Cell cycle distribution was analyzed using FlowJo software (Tree Star, Inc., Ashland, OR).

### 2.7 Intracellular ROS measurement

Reactive oxygen species (ROS) production was investigated as a potential cytotoxic mechanism induced by the *Attalea butyracea* crude extract in *G. lamblia*. Flow cytometry (FACS Canto II flow cytometer, Becton & Dickinson, New Jersey, NY) was employed to quantify ROS levels using the fluorescent probe 2′,7′-dichlorodihydrofluorescein diacetate (H_2_DCFDA), which oxidizes to 2′,7′-dichlorofluorescein (DCF) in the presence of ROS, emitting fluorescence proportional to the oxidative capacity of reactive species. ROS levels were measured in *G. lamblia* WB/1267 trophozoites treated for 24 h with or without the crude extract using Image-iT LIVE Green Reactive Oxygen Species Detection Kit (Invitrogen, Massachusetts, United States), following the manufacturer’s protocol. Additionally, ROS formation was visualized using confocal microscopy (Olympus FV1200). Image processing was conducted using the Fiji Image software package.

### 2.8 Indirect immunofluorescence assays and confocal microscopy

Antibodies against tubulin (1:500; Sigma-Aldrich Co.) and monoclonal antibodies targeting PVs (Rivero et al., 2010) were employed to investigate changes in the cytoskeleton and assess alterations in the distribution and morphology of PVs, respectively. *G. lamblia* WB/1267 trophozoites were fixed in 4% v/v formaldehyde solution in 1X PBS for 40 min at room temperature. Blocking was performed using a solution containing 3% w/v bovine serum albumin (BSA, Sigma-Aldrich Co., United States) and 0.05% v/v Tween (Sigma-Aldrich Co., United States) in 1X PBS. Subsequently, cells were incubated with the primary antibody diluted in 1.5% w/v BSA and 0.05% v/v Tween in 1X PBS. After washing three times with 0.05% v/v Tween in 1X PBS, cells were exposed to Alexa Fluor 488-conjugated secondary antibodies (dilution 1:500, Life Technologies) diluted in 1.5% w/v BSA and 0.05% v/v Tween in 1X PBS for 1 h at 37°C in a humid chamber. The following three washes were done with 1X PBS, and the cells were stained with DAPI for 5 min. Samples were mounted using FluorSave mounting medium (Merck Group, Darmstadt, Germany) and visualized with an Olympus FV1200 confocal microscope. Image analysis was performed using Fiji software^2^ and Adobe Photoshop 8.0 (Adobe Systems).

### 2.9 Ultrastructure analysis

Transmission Electron Microscopy (TEM) was utilized to analyze the ultrastructure of *G. lamblia* trophozoites WB/1267 following exposure to *Attalea butyracea* fruit extract. Trophozoites treated with IC_50_ and 2 × IC_50_ concentrations of crude extract for 48 h, along with untreated controls, were fixed in a solution containing 4% v/v formaldehyde and 2% v/v glutaraldehyde in 0.1 M cacodylate buffer and stored at 4°C. Subsequently, the fixed cells underwent centrifugation, and the resulting pellets were washed and treated with 1% v/v osmium tetroxide for 1 h, followed by dehydration in a graded series of cold acetones. The samples were embedded in Spur resin and polymerized at 60°C for 48 h. Thin sections (90 nm thick) were cut using an RMC Power Tome-XL ultramicrotome and examined for ultrastructural changes, focusing on nuclei, flagella, ventral disks, PVs, chromatin, and cellular morphology using a Hitachi HT 7800 electron microscope (Hitachi, Tokyo, Japan).

### 2.10 Synergism of metronidazole and *Attalea butyracea* fruit extract

*G. lamblia* WB/1267 trophozoites were used to check MTZ and *Attalea butyracea* fruit extract interaction. For combination tests, dilutions were made using the fixed concentration method, where the IC_50_ of MTZ was kept constant and the crude extract was diluted in constant fractions of its IC_50_. The fractional inhibitory concentration index (FICI) was estimated using the following formula: FIC_A_ + FIC_B_ = FICI, where FIC_A_ is the value of IC_50_ *Attalea butyracea* fruit extract in the combination/IC_50_ value of *Attalea butyracea* fruit extract alone and FIC_B_ is the value of IC_50_ MTZ in the combination/IC_50_ value of MTZ alone (Chamberland, 1993). The interaction was classified as synergy if the FICI was ≤0.5, additivity if the FICI ranged from 0.5 to 1, antagonism if the FICI was >4.0, and no interaction if FICI was between 1 and 4.0 (Odds, 2003; Mulyaningsih et al., 2010)_._

### 2.11 Statistical analyses

Data are expressed as the mean ± standard error of the mean (SEM). Statistical analysis was performed using one-tailed paired and unpaired Student’s *t*-tests, as applicable, with GraphPad Prism 9 (GraphPad Software Inc., United States^3^). *p*-values ≤ 0.05 were considered statistically significant. All experiments were carried out in triplicate and independently repeated at least three times.

## 3 Results

### 3.1 *In vitro* activity of plant extracts from the Colombian Amazon against *Giardia lamblia* trophozoites

**Figure 1:**
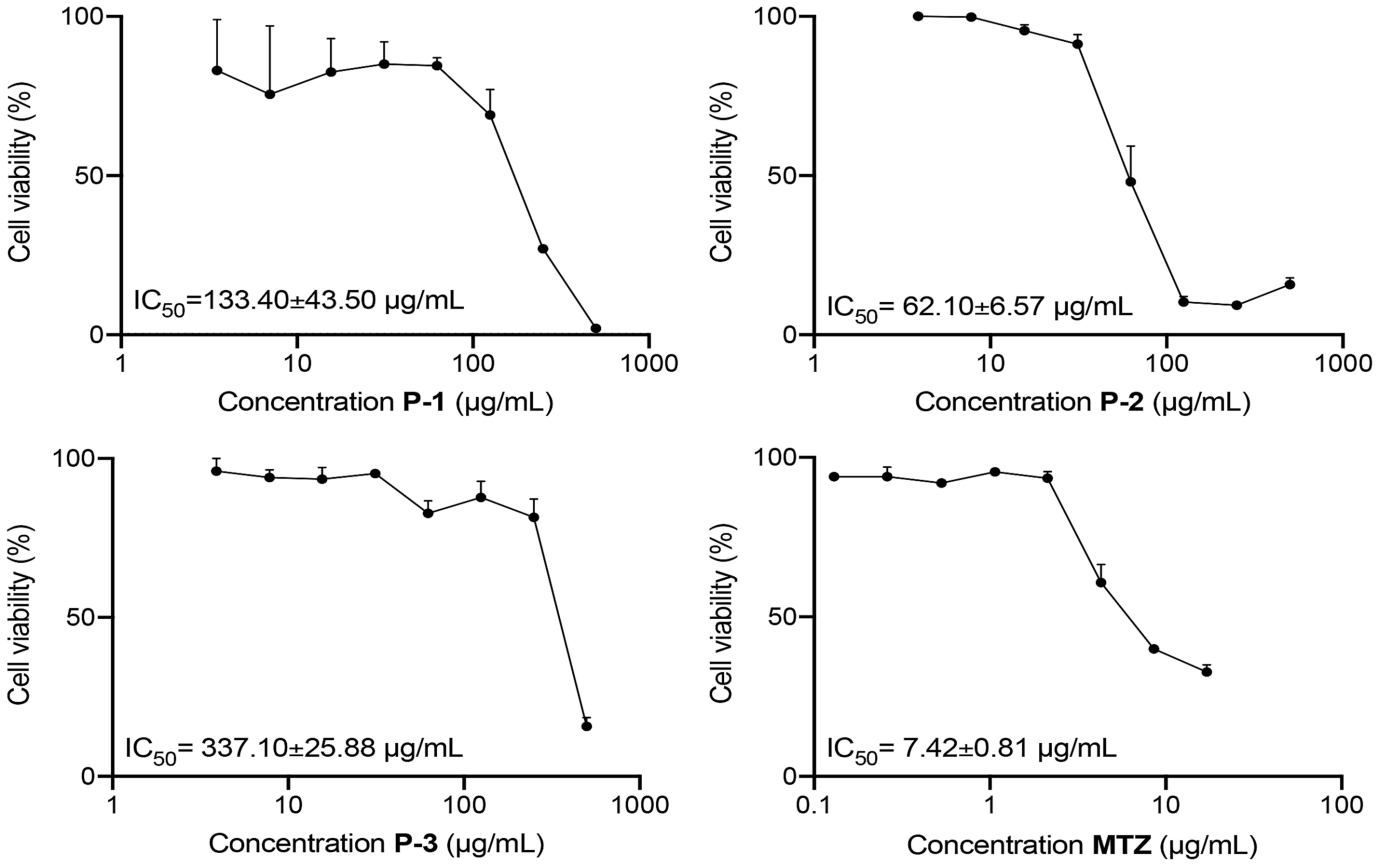
Dose–response curves for the cytotoxicity of **P-1**, **P-2**, and **P-3** crude extracts and **MTZ** on *G. lamblia* WB/1267 trophozoites, after treatment for 48 h. Values are expressed as mean ± standard error (SEM) from at least three independent experiments.

In the initial screening, the giardicidal activity of 15 crude extracts was evaluated at a concentration of 500 μg/mL against *G. lamblia* WB/1267 trophozoites. Among these extracts, those from *Astrocaryum chambira* (**P-1**), *Attalea butyracea* (**P-2**), and *Bactris gasipaes* (**P-3**) showed significant activity, with cytotoxicity percentages exceeding 80% (Table 1). Subsequently, IC_50_ values were calculated based on these cytotoxicity results. The WB/1267 trophozoites responded to the extracts in a dose-dependent manner. Based on the criteria proposed by Amaral et al. (2006), a compound is considered highly active when IC_50_ ≤ 100 μg/mL, active when 100 < IC_50_ ≤ 250 μg/mL, moderately active when 250 < IC_50_ ≤ 500 μg/mL, and inactive when IC_50_ ≥ 500 μg/mL. After 48 h of treatment, the **P-2** extract exhibited the highest giardicidal efficacy with an IC_50_ of 62.10 ± 6.57 μg/mL, indicating potent activity. The **P-1** and **P-3** extracts followed with IC_50_ values of 133.40 ± 43.50 μg/mL (active), and 337.10 ± 25.88 μg/mL (moderately active), respectively (Figure 1). Additionally, the IC_50_ of MTZ, a first-line drug for giardiasis treatment, was determined as positive control, showing an IC_50_ of 7.42 ± 0.81 μg/mL.

**Table 1:** Cytotoxicity of plant extracts from the Colombian Amazon against *Giardia lamblia* WB/1267 trophozoites.

Plant extract	Scientific name	Part used	Traditional plant uses	References	Giardicidal activity (500 μg/mL)
**P-1**	*Astrocaryum chambira* (Burret)	Fruit	DI	Valencia et al. (2013) and Mejía (2016)	94.5 ± 0.5%
**P-2**	*Attalea butyracea* (Mutis ex. L.f.) Wess. Boer	Fruit	DIA - DI - GI	Alexiades (1999) and Balslev et al. (2008)	96.5 ± 0.5%
**P-3**	*Bactris gasipaes* (Kunth)	Fruit	StP - DI	Mejía (2016) and González-Jaramillo et al. (2022)	89.5 ± 0.5%
**P-4**	*Cecropia ficifolia* (Löfling)	Leaf	DI	Paniagua Zambrana and Bussmann (2017)	Inactive
**P-5**	*Inga edulis* (Mart)	Fruit	GI	Lima et al. (2020)	Inactive
**P-6**	*Mangifera indica* (Linneo)	Fruit	DI - DIA - GI	Shah et al. (2010)	Inactive
**Р-7**	*Mangifera indica* (Linneo)	Leaf	DI - DIA - GI	Shah et al. (2010) and Kujawska and Schmeda-Hirschmann (2022)	Inactive
**P-8**	*Manihot esculenta* (Crantz)	Fruit	GI	Kuete (2014)	Inactive
**P-9**	*Manihot esculenta* (Crantz)	Leaf	StP - DIA - GI	Bahekar and Kale (2013) and Kuete (2014)	Inactive
**P-10**	*Mauritia flexuosa* (Linneo)	Leaf	DIA - GI	Menezes de Oliveira et al. (2022)	Inactive
**P-11**	*Musa* sp. (Linneo)	Fruit	DIA - GI	Karuppiah and Mustaffa (2013)	Inactive
**P-12**	*Psidium guajava* (Linneo)	Fruit	DIA - GI	Gutiérrez et al. (2008) and Gutierrez-Montiel et al. (2023)	Inactive
**P-13**	*Psidium guajava* (Linneo)	Leaf	GI	Gutiérrez et al. (2008)	Inactive
**P-14**	*Renealmia alpinia* (Rottb)	Fruit	StP	Gómez-Betancur et al. (2015)	Inactive
**P-15**	*Zygia longifolia* (Humb. & Bonpl. ex Willd.)	Leaf	GI	De la Torre et al. (2008)	12 ± 1%

StP, Stomach Pain; DI, Digestive Issue; DIA, Diarrhea; GI, Gastrointestinal Disorders.

### 3.2 Assessment of apoptosis and cell cycle distribution

**Figure 2:**
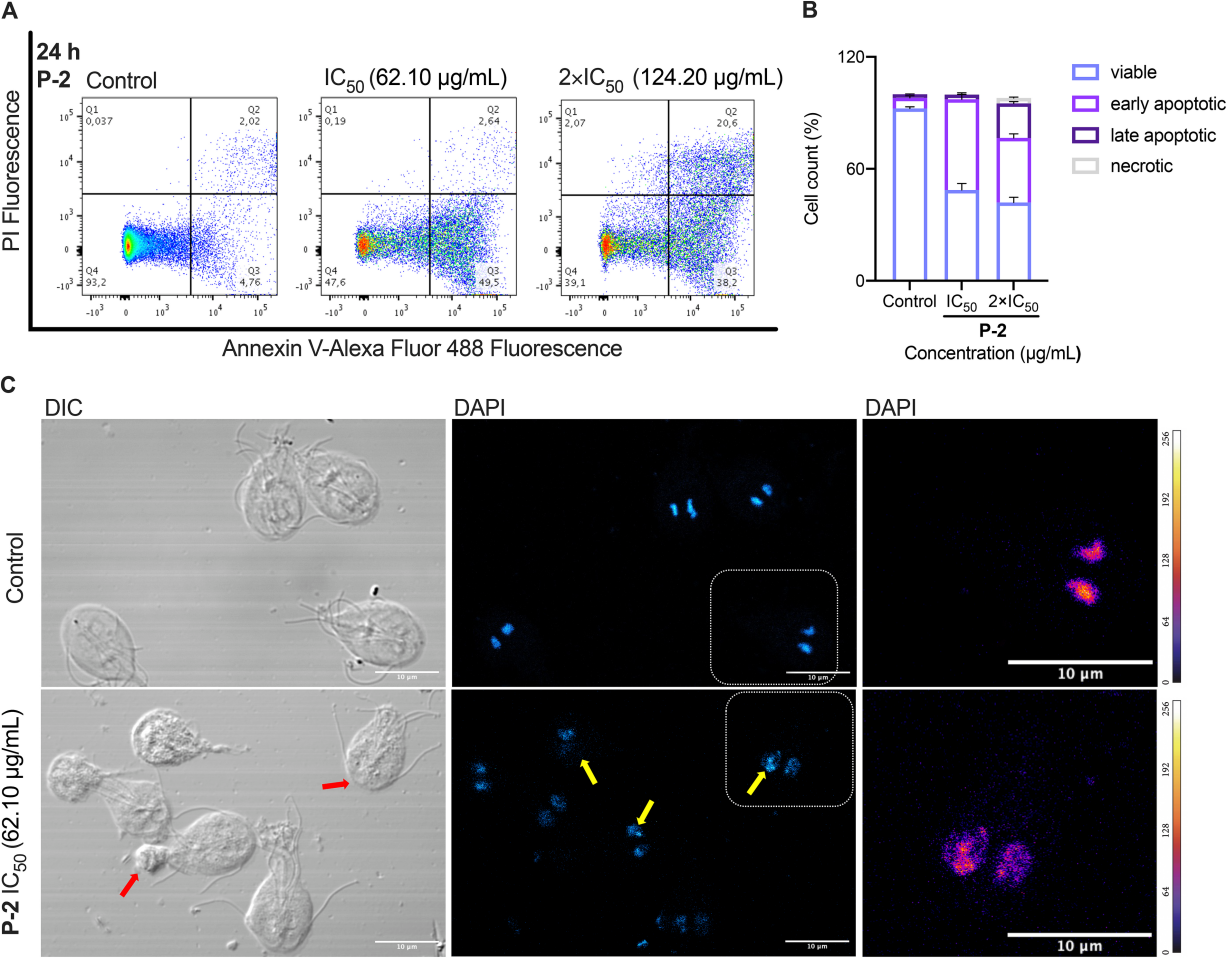
Apoptotic and necrotic effects of **P-2** treatment on *G. lamblia* trophozoites. **(A)** Flow cytometry analysis of *G. lamblia* trophozoites treated with **P-2** crude extract for 24 h at concentrations equivalent to IC_50_ and 2 × IC_50_, stained with Annexin V-FITC to assess apoptosis. Data shows one representative of triplicate independent experiments. **(B)** Quantification of viable, early apoptotic, late apoptotic, and necrotic cells based on flow cytometry analysis. **(C)** Fluorescence microscopy using DAPI staining confirmed apoptotic features such as chromatin condensation and nuclear fragmentation (yellow arrows). Differential interference contrast (DIC) microscopy revealed membrane buddings (red arrows), likely corresponding to apoptotic bodies. Heatmap representation of DAPI pixel intensity for the selection shown in DAPI. The bar shows heatmap index. Bars: 10 μm. Data shows one representative of triplicate independent experiments.

The promising giardicidal activity exhibited by the **P-2** prompted its selection as a candidate for investigating its mechanism of cell death. Cells treated for 24 h with the crude extract at concentrations equivalent to IC_50_ and 2 × IC_50_ were stained with Annexin V- Alexa Fluor^®^ 488 to assess apoptosis. Flow cytometry analysis (Figure 2A) showed increased apoptosis, indicated by enhanced fluorescence. Quantification of the data (Figure 2B) revealed a significant reduction in viable cells in the treated groups compared to the untreated control (92.30 ± 0.90% vs. 48.63 ± 3.47%, *p* < 0.01, and 41.90 ± 2.57%, *p* < 0.01, respectively). In parallel, there was a marked rise in early apoptotic cells (48.67 ± 3.40 and 34.67% ± 2.14%, *p* < 0.01, respectively) and late apoptotic cells (2.49 ± 0.08%, *p* < 0.05, and 18.43 ± 1.09%, *p* < 0.001). The increase in late apoptotic cells was significantly more pronounced with the 2 × IC_50_ treatment (*p* < 0.0001), demonstrating a dose-dependent effect of **P-2** on *G. lamblia* trophozoites. Additionally, a significant rise in necrotic cells was observed (0.16 ± 0.03%, *p* < 0.05, and 2.96 ± 0.47%, *p* < 0.01, vs. control 0.03% ± 0.01%), as determined by propidium iodide uptake (Figures 2A,B).

The apoptotic and necrotic effects observed were further confirmed through morphological changes observed under fluorescence microscopy using DAPI staining (Figure 2C). In contrast to the control, treatment with **P-2** triggered typical apoptotic features such as condensed chromatin. Differential interference contrast (DIC) microscopy revealed the presence of membrane blebbing emerging from the trophozoite, consistent with previous descriptions following treatment with MTZ and furazolidone (Campanati and Monteiro-Leal, 2002). These structures may correspond to the formation of apoptotic bodies.

**Figure 3:**
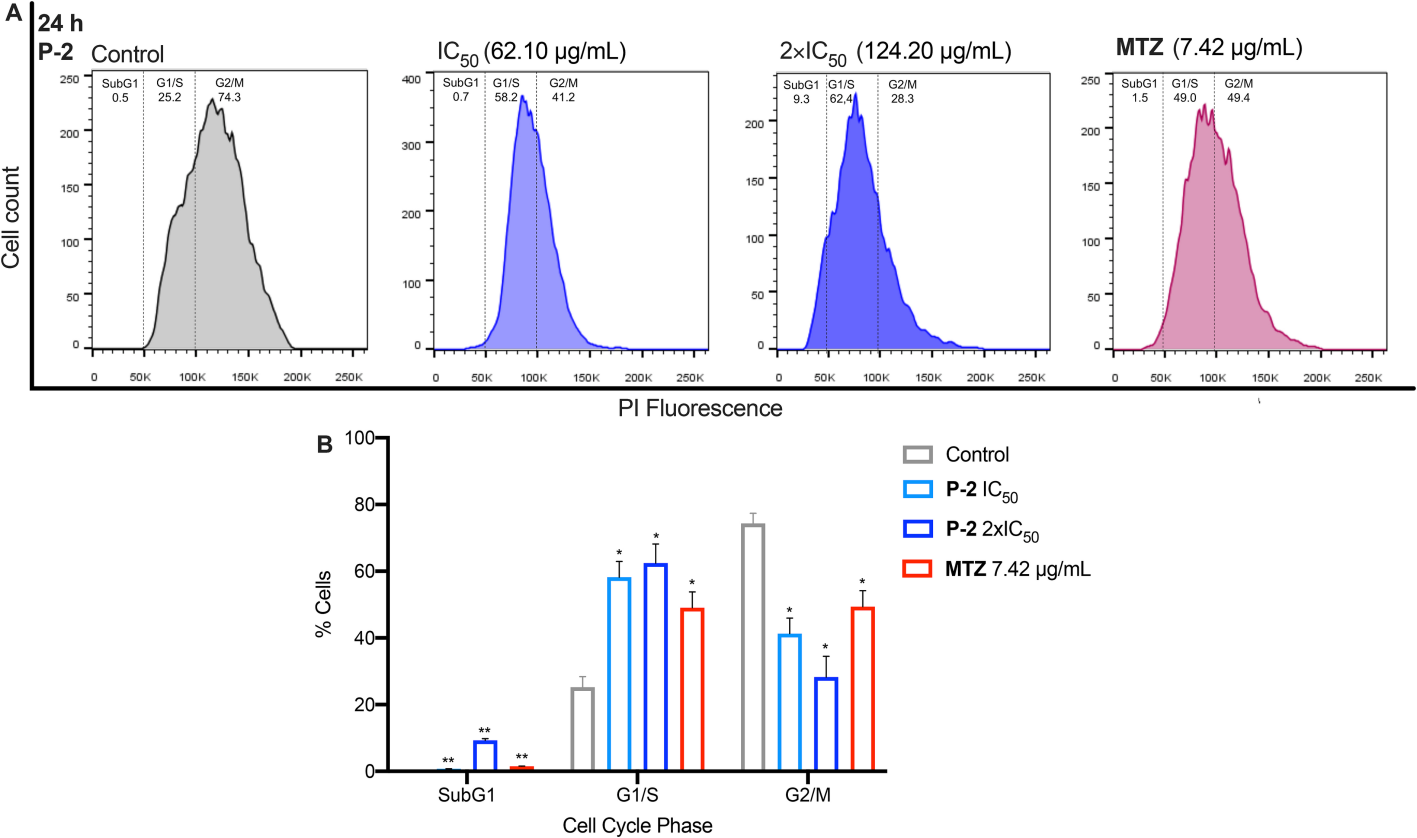
The effects of **P-2** on cell cycle progression in *G. lamblia* trophozoites. **(A)** The impact of **P-2** at IC_50_ and 2 × IC_50_ concentrations, as well as MTZ, on the cell cycle. Following labeling with propidium iodide (PI) staining solution, the samples were analyzed by flow cytometry. Panel **(A)** shows a representative experiment from three independent trials. **(B)** The percentage of cells in each phase of the cell cycle is displayed. Bars represent means ± SEM. Differences were analyzed using one-tailed unpaired *t*-tests (**p < 0.01, *p < 0.05).

Further flow cytometry analyses were conducted to investigate how **P-2** disrupts cell cycle progression in *G. lamblia* trophozoites (Figures 3A,B). In untreated control cells, the maximum number of cells were found in G2/M phase (74.30 ± 3.10%) with a subset in the G1/S phase (25.25 ± 3.15%). Exposure of trophozoites to **P-2** at IC_50_ and 2xIC_50_ resulted in a marked reduction of the G2/M subpopulations (41.20 ± 4.70% *p* < 0.05 and 28.30 ± 6.20% *p* < 0.05, respectively), with a significative increase in the G1/S subpopulations (58.15 ± 4.75 *p* < 0.05 and 62.45 ± 5.75 *p* < 0.05, respectively). Simultaneously, a significant increase in cells at the SubG1 subpopulation in accordance with the presence of cells undergoing apoptosis was observed (*p* < 0.01) (Figures 3A,B). Trophozoites treated with MTZ were simultaneously processed as a positive control. This drug arrested the cells in the G1/S phases (49.05 ± 4.75 *p* < 0.05), consistent with earlier findings (Uzlikova and Nohynkova, 2014). The effect of MTZ was associated with a significant increase (*p* < 0.01) in the SubG1 subpopulation resembling the results obtained at both concentrations of **P-2**. These results collectively suggest that cytotoxic concentrations of **P-2** induced a cell cycle arrest at the G1/S phases, with a substantial reduction of parasites at the G2 phase, leading to apoptosis which is reflected in the increase of the SubG1 subsets.

### 3.3 Examination of ROS generation

**Figure 4:**
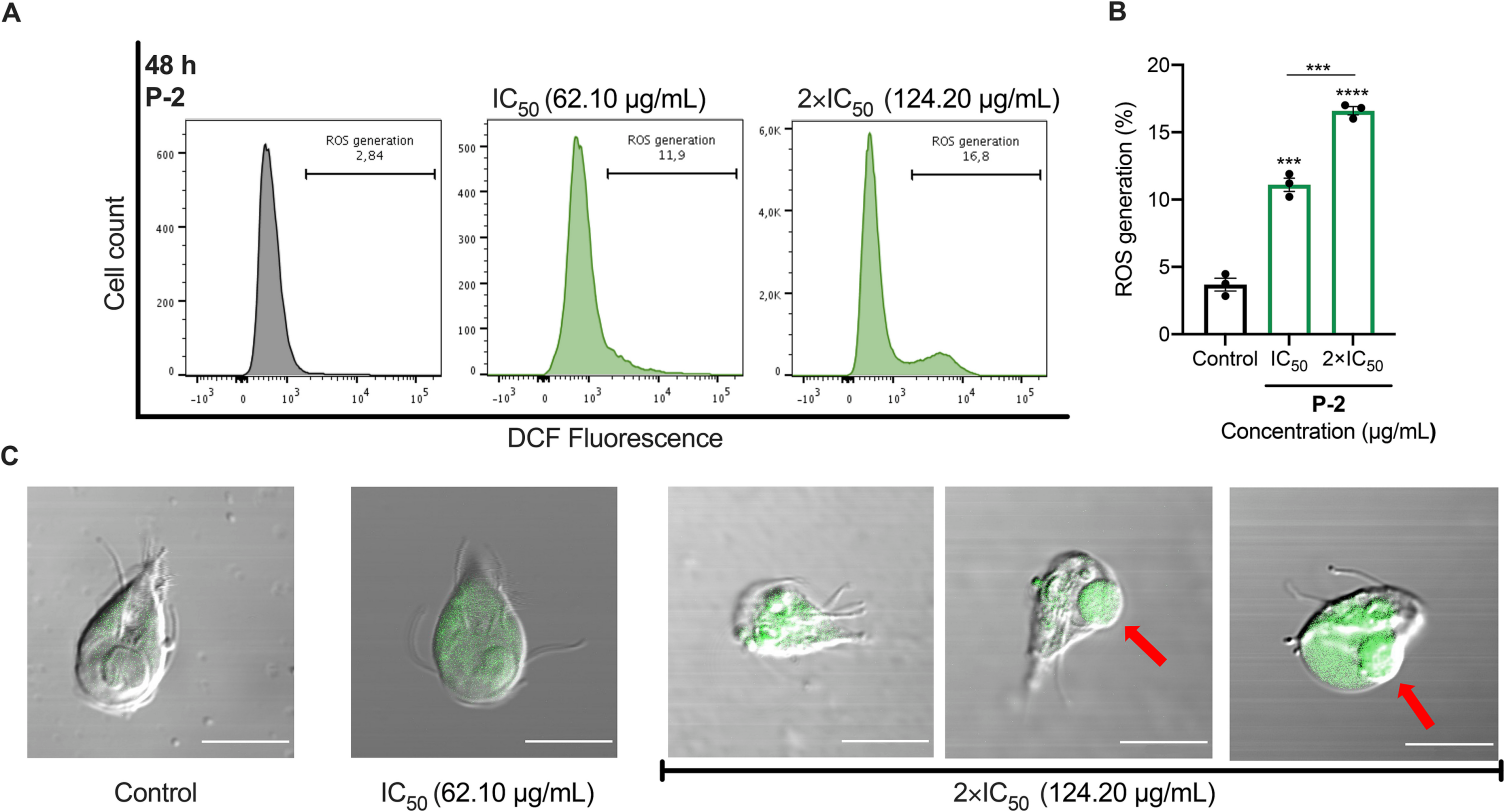
ROS generation and oxidative stress induced by **P-2** in *G. lamblia* trophozoites. **(A)** ROS production was assessed in *G. lamblia* WB/1267 trophozoites treated with **P-2** using DCF-DA staining and analyzed by flow cytometry. The figure shows a representative result from three independent experiments. **(B)** Graph showing the percentage of ROS generation. Treated trophozoites exhibited significantly increased ROS levels compared to untreated controls. Bars represent means ± SEM. Differences were analyzed using one-tailed unpaired *t*-tests (***p < 0.0001, ***p < 0.001). **(C)** DIC-merged confocal fluorescence microscopy showing ROS distribution in the cytoplasm of *G. lamblia* WB/1267 trophozoites. Membrane blebbing (red arrows) were observed. DCF, highly fluorescent dichlorofluorescein; DIC, Differential interference contrast. Bars 10 μm.

We investigated the connection between apoptosis-like cell death induced by **P-2** and oxidative stress. Flow cytometry analysis showed that *G. lamblia* trophozoites treated with IC_50_ and 2 × IC_50_ concentrations of **P-2** exhibited significantly increased levels of ROS. The values were 11.10 ± 0.49% (*p* < 0.001) and 16.60 ± 0.30% (*p* < 0.0001), respectively, compared to the control group (3.69 ± 0.47%). Notably, treatment with 2 × IC_50_ resulted in a 1.5-fold increase in ROS production compared to IC_50_ treatment (*p* < 0.001) (Figures 4A,B). Confocal fluorescence microscopy, used to map the intracellular distribution of ROS in the cytoplasm of WB/1267 trophozoites treated with IC_50_, revealed a predominantly punctate pattern in most cells (Figure 4C). In cells exposed to 2 × IC_50_, pronounced morphological changes were observed, including the formation of prominent membrane blebbing densely packed with ROS (Figure 4C). The enhanced ROS accumulation underscores the intensified oxidative stress under higher treatment conditions.

### 3.4 Evaluation of ultrastructural damages on P-2 treated cells

**Figure 5:**
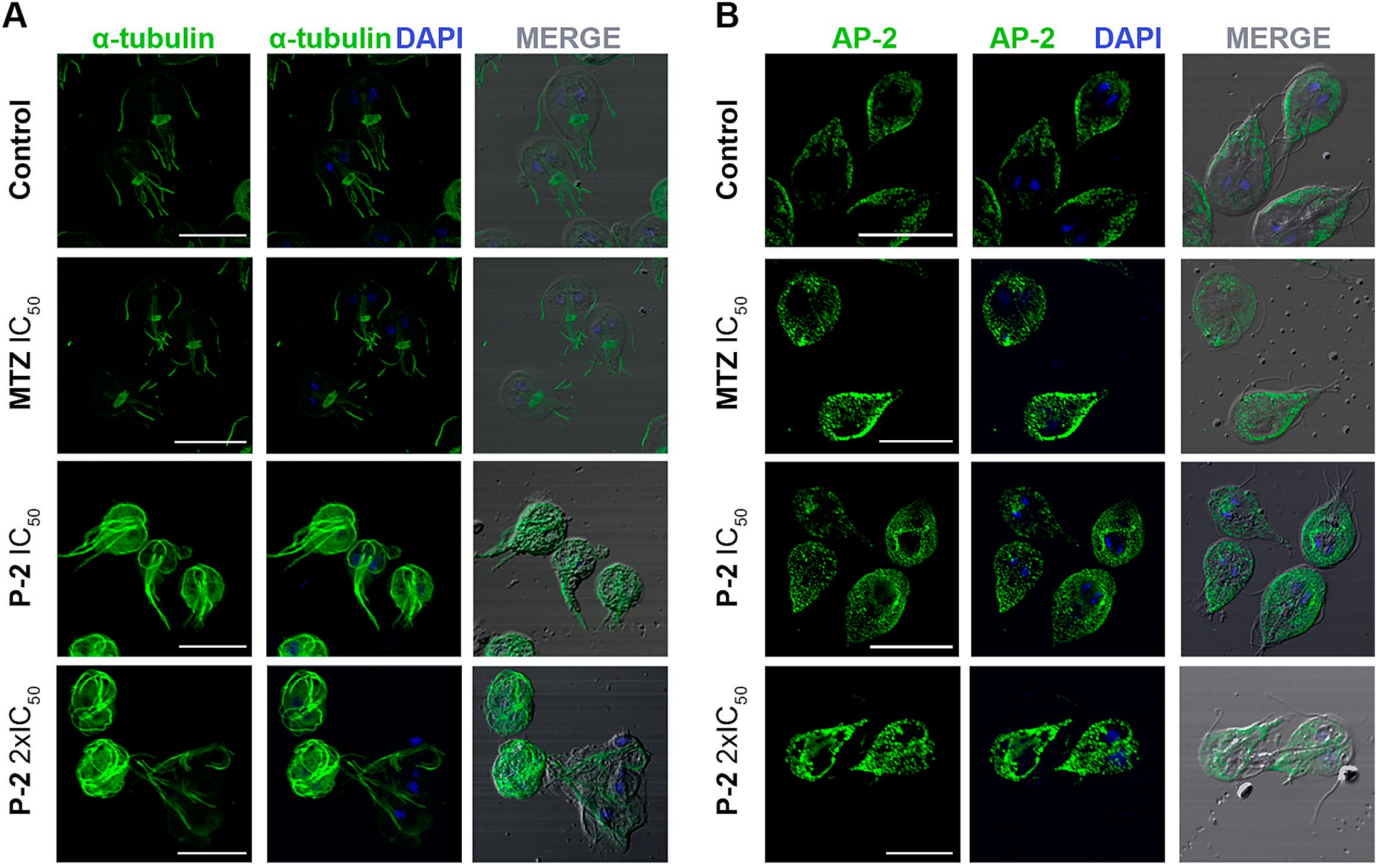
Effect of **P-2** treatment on cytoskeletal and peripheral vacuole alterations in *Giardia lamblia* trophozoites. **(A)** Immunofluorescence analysis and confocal microscopy of *G. lamblia* trophozoites treated with **P-2** reveal significant cytoskeletal alterations. Alpha-tubulin was labeled with a monoclonal antibody to visualize microtubule structures such as the ventral disk, flagella, and median body. **(B)** Immunofluorescence and confocal microscopy of peripheral vacuoles (PVs) using AP-2 subunit Glμ2 (5E2) MAb show that **P-2** treatment caused a significant alteration in PV distribution, with increased cytoplasmic localization compared to the untreated control. DIC: Differential interference contrast. MTZ: metronidazole. Bars 10 μm.

An analysis of *G. lamblia* trophozoites revealed significant structural alterations in response to **P-2** treatment. To assess cytoskeletal changes, we utilized alpha-tubulin labeling using a monoclonal antibody (Mab) to visualize the organization of microtubules into structures such as the ventral disk, flagella, and median body. The IFA demonstrated a pronounced and abnormal redistribution of tubulin compared to the control, with effects becoming more evident at 2 × IC_50_ (Figure 5A). Significant morphological changes were also observed, including the formation of rounded cells and retraction of the flagella (Figure 5A). The alteration in the cytoskeleton induced by **P-2** differs from the changes caused by MTZ at IC_50_. Specifically, IFA results showed no significant changes in the cytoskeleton under MTZ treatment compared to the control. Thus, the impact of **P-2** on the alpha-tubulin is distinct from that of MTZ, suggesting differences in their mechanisms of action.

Other critical structures to investigate are the peripheral vacuoles (PVs), which are polarized and located beneath the plasma membrane on the dorsal side of the trophozoite. The PVs in *Giardia* play a role in acidification and digestion, functioning similarly to early and late endosomes or lysosomes in other organisms (Lanfredi-Rangel et al., 1998; Rivero et al., 2013). The multifunctionality of PVs indicates that they are key organelles in the analysis of how drugs are processed and how they can affect their metabolic function. For this, a monoclonal MAb against the medium subunit of clathrin-adaptor protein (Glμ2) (Rivero et al., 2010) was used to analyze the effect of **P-2** in the PVs distribution. It was observed that **P-2** treatment induced significant alteration of the labeling beneath the plasma membrane when compared with the untreated control. It was reported by using transmission electron microscopy (TEM), which revealed that trophozoites treated with MTZ exhibited changes in the size, content, and localization of the PVs (Benchimol et al., 2023). Similarly, in this study, when the PVs were analyzed by IFA and confocal microscopy, changes in the area occupied by the PVs were increased when MTZ was added at its IC_50_, comparing with the untreated control, supporting the findings described using TEM (Figure 5B) (Benchimol et al., 2023). Likewise, **P-2**-treated trophozoites showed an increase in PVs’ size and pronounced cytoplasmic localization (Figure 5B). These results indicate a substantial alteration in the dynamics and distribution of PVs within the cytoplasm at higher treatment concentrations, suggesting disruptions in estandar endo-lysosomal processing.

**Figure 6:**
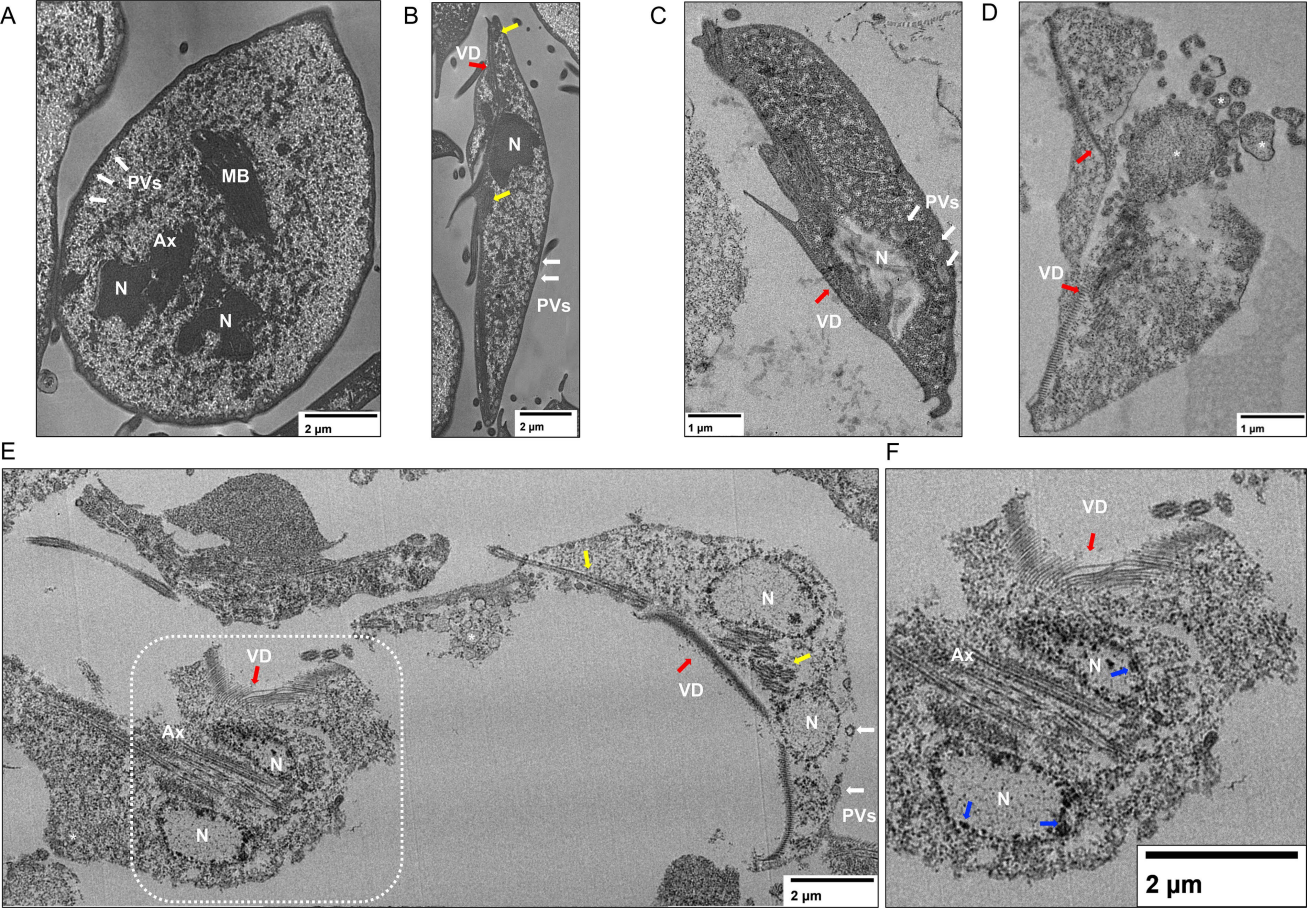
Ultrastructural analysis of *Giardia lamblia* trophozoites treated with **P-2** by transmission electron microscopy (TEM). **(A,B)** TEM images of untreated *G. lamblia* trophozoites show intact ultrastructure, including the ventral disk and flagella. **(C,D)** Trophozoites treated with **P-2** at IC_50_ displayed an enlargement of the peripheral vacuoles (PVs, white arrows) and membrane blebbing (asterisks). **(E)** The 2 × IC_50_ of **P-2** treatment resulted in advanced cellular damage and structural disintegration. **(F)** Amplification of demarked inset on E shows chromatin condensations in detail. N, nucleus; MB, Medial body; VD, ventral disk (red arrows); Ax, axoneme. Yellow arrows denote the flagella on different planes. Blue arrows demark chromatin condensations. Bars’ sizes are denoted in each picture.

When the ultrastructure alterations of **P-2** treatment were analyzed further by TEM, significant changes in the ventral disk were observed at higher concentrations. However, the regularly spaced spiral microtubule composition was preserved in part. No significative changes in the flagella ultrastructure were observed between untreated (Figures 6A,B) and treated trophozoites (Figures 6C–F). Also, the *Giardia* axonemes, which have a lengthy cytosolic portion before becoming active flagella, were unaffected. However, the PVs’ sizes increased, and membrane blebbing appeared at IC_50_ (Figures 6C,D). Under treatment with 2 × IC_50_, the presence of chromatin condensation and emptiness of the cytoplasm is the predominant condition of these trophozoites (Figures 6E,F). These findings suggest that **P-2** treatment induces significant ultrastructural changes, particularly at higher concentrations, affecting cellular integrity and vacuolar dynamics.

### 3.5 Synergistic effect of P-2 and metronidazole on *Giardia lamblia* WB/1267 trophozoites

**Figure 7:**
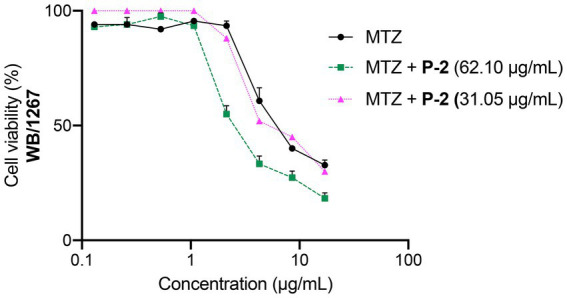
Dose–response curves for the cytotoxicity of MTZ at its IC_50_ (7.42 ± 0.81 μg/mL) alone and in combination with IC_50_ (62.50 μg/mL) and to ½ *×* IC_50_ (31.25 μg/mL) of **P-2** on *G. lamblia* WB/1267 trophozoites. Values are expressed as mean ± SEM from at least three independent experiments.

The abbreviated diagonal sampling checkerboard methodology was employed to determine how **P-2** interacts with MTZ and find potential synergistic combinations (Odds, 2003). The fractional inhibitory concentration index (FICI) was estimated using the following formula: FICA + FICB = FICI, where FICA is the value of **P-2** in the combination/value of **P-2** alone, and FICB is the value of MTZ in the combination/value of MTZ alone. The interaction was classified as “synergy” if FICI ⩽0.5, “additivity” if FICI = 0.5–1.0, “antagonism” if FICI >4.0, and “no interaction” if FICI ≥ 1.0 < 4.0. The combination of the **P-2** extract at the IC_50_ (62.10 μg/mL) with the IC_50_ (7.42 ± 0.81 μg/mL) of MTZ resulted in a synergistic effect (FICI = 0.32 ± 0.01), enhancing the overall antiparasitic activity (Figure 7). This synergy was observed through a notable decrease in trophozoite viability and increased inhibition of growth compared to treatment with MTZ alone (MTZ in combination IC_50_ = 2.37 ± 0.26 vs. MTZ alone IC_50_ = 7.42 ± 0.81 μg/mL, *p* < 0.01). When the extract concentration was reduced to ½ *×* IC_50_, there was no interaction with MTZ since the activity remained unchanged (FICI = 1.18 ± 0.08, Figure 7). These results indicate synergistic activity for **P-2** at the IC_50_ in combination with MTZ, opening promising possibilities for dose reduction during the treatment of giardiasis and for resistant isolates.

### 3.6 Activity of *Attalea butyracea* fruit extract against *Giardia lamblia* GS/M trophozoites

**Figure 8:**
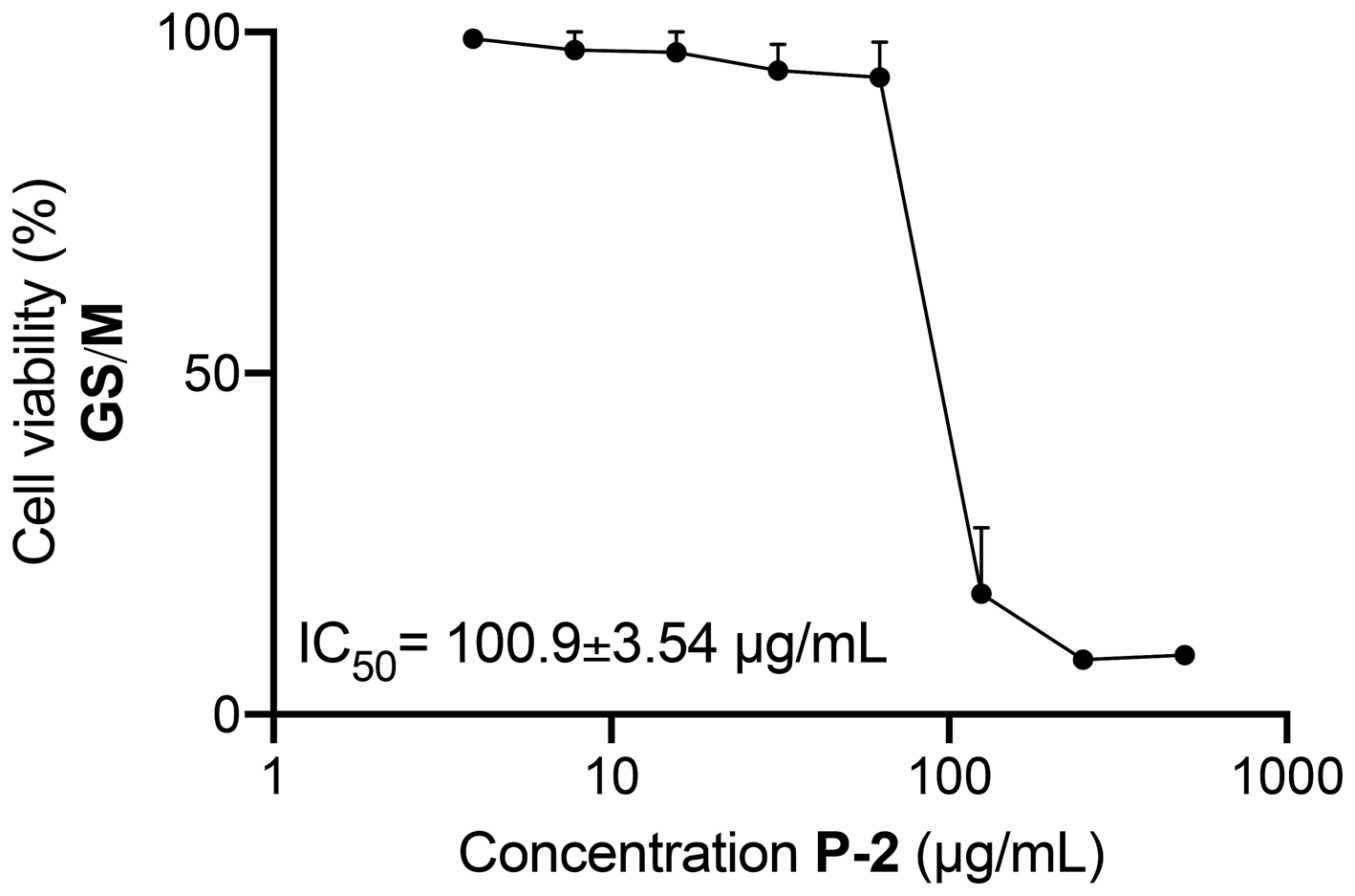
Inhibitory activity of *Attalea butyracea* fruit extract (**P-2**) against *Giardia lamblia* GS/M trophozoites (genotype B). The graph illustrates the dose–response curve of the **P-2** extract, showing a reduction in the viability of GS/M trophozoites with increasing concentrations. The efficacy of **P-2** was significant compared to other strains, showing a slightly higher IC_50_ value than that observed in WB/1267 strains (*p* < 0.01). Data points represent mean values ± SE from three independent experiments.

Genotypes of *Giardia lamblia* show differing levels of host specificity, with only genotypes A and B capable of infecting humans (Cacciò et al., 2018). In the gerbil model, genotype B caused more pronounced pathogenic effects, including more significant intestinal damage and higher trophozoite loads, compared to genotype A (Bénéré et al., 2012). Pioneering human studies also confirmed that genotype B isolates are more infectious and virulent, showing higher rates of symptomatic infection and variability based on antigen-expressing clones (Nash et al., 1987, 1990). Given the strains’ drug resistance and pathogenicity variability, the extract’s efficacy was tested against *G. lamblia* GS/M trophozoites (genotype B). The extract, **P-2**, demonstrated significant antiparasitic activity, inhibiting GS/M trophozoite growth with an IC_50_ of 100.90 ± 3.40 μg/mL (Figure 8), slightly higher than the IC_50_ reported for the WB/1267 strains.

### 3.7 Chromatographic profile of *Attalea butyracea* fruit extract

**Figure 9:**
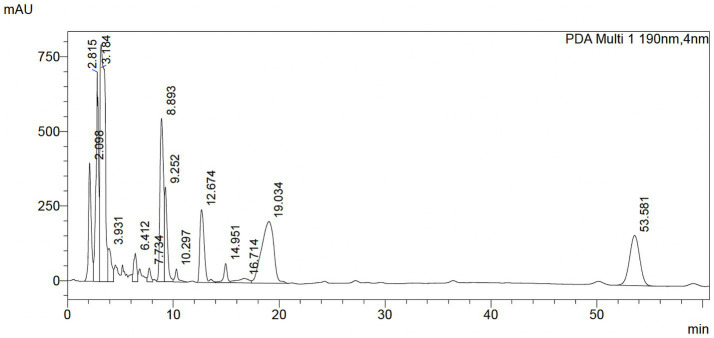
HPLC profile of *Attalea butyracea* fruit extract dissolved in ethanol. The mobile phase was acetonitrile/water 90:10 and UV detection at 190 nm.

The qualitative HPLC fingerprint profile of the hydroethanolic **P-2** extract was obtained at a wavelength of 190 nm. Several distinct peaks with varying retention times were observed. Notably, eight prominent peaks were displayed at a retention time of 2.098, 2.815, 3.184, 8.893, 9.252, 12.674, 19.034, and 53.581 min (Figure 9). These peaks are likely to correspond to different chemical compounds present in the extract, reflecting the complex nature of the sample. The distribution of peaks indicates the presence of both low- and high-molecular-weight components, which may vary in polarity and functional groups. A comprehensive table containing the retention times, areas, and heights of the observed peaks is provided in the Supplementary Table S2.

## 4 Discussion

The fruit of certain species has served as a vital dietary component for indigenous communities throughout South America across the ages. From time-honored remedies passed through generations to dietary mainstays supporting communities, these plants offer a rich repository of potential solutions to combat diverse pathologies. After evaluating the giardicidal activity of 15 crude plant extracts from the Colombian Amazon, the *Attalea butyracea* fruit extract (**P-2**) demonstrated the highest efficacy, with significant activity against *Giardia* strains WB/1267 and GS/M. Notably, **P-2** also demonstrated synergistic activity with metronidazole, enhancing its therapeutic potential against giardiasis.

*Attalea butyracea*, commonly known as the “wine palm,” has a range of ethnopharmacological applications, particularly in traditional medicine across South and Central America. Indigenous groups have historically used various parts of the plant, including the fruit, leaves, and oil extracted from its seeds, for medicinal and practical purposes (Bernal et al., 2010). Regarding ethnopharmacological applications, the fruit and oil of *Attalea butyracea* have been traditionally employed to treat gastrointestinal illnesses, such as diarrhea and stomach pain, much like many other palm species in indigenous cultures (Alexiades, 1999; da Silveira Agostini-Costa, 2018). Notably, many of these gastrointestinal disorders are associated with parasitic infections, including those caused by *Giardia* species. In this context, the traditional use of *Attalea butyracea* to alleviate intestinal discomfort is particularly significant, suggesting its potential as an antiparasitic agent. However, to date, no scientific evidence has been reported regarding its mechanism of action.

While these traditional uses are ancient and recognized by these communities, the discovery of its antiparasitic activity, particularly against *Giardia lamblia*, adds a novel dimension to the plant’s therapeutic potential and suggests that it may help address protozoal infections alongside its established roles in treating digestive disorders.

The treatment of giardiasis primarily relies on MTZ, which has been widely used for over 50 years. While its efficacy ranges from 73 to 100%, growing clinical resistance and significant side effects, such as headaches, nausea, and more severe issues like pancreatitis and peripheral neuropathy, contribute to treatment failure (Campanati and Monteiro-Leal, 2002; Saghaug et al., 2019; Riches et al., 2020). Its mode of action involves the activation of nitro radicals that damage parasite DNA, disrupting its cell cycle. In this study, the antiparasitic effect of **P-2** was primarily through the induction of early apoptosis in *Giardia* WB/1267 trophozoites, progressing to late apoptosis and necrosis at higher concentrations, like MTZ, **P-2** caused chromatin condensation, cell cycle arrest, and oxidative stress in the parasites. Conversely to MTZ, the structural damage induced by **P-2** included cytoskeletal disorganization. However, the tubulin-specific Mab still in IFA recognizes the tubulin proteins, indicating that **P-2** altered the cytoskeleton organization rather than the tubulin itself. These results were corroborated by TEM in which the trophozoites treated with 2 × IC_50_ of **P-2** were misshapen and showed a complete emptiness of the cytoplasm and the ventral disk fragmentation, as was described after treatment with 1 μg/mL of ABZ for 24 h (Benchimol et al., 2023). On the other hand, the effect of **P-2** on the size and depolarization of the endo-lysosomal PVs has been observed in cells treated with different concentrations of MTZ (Benchimol et al., 2023). These effects on the PVs might be associated with the direct impact of ROS production over their membranes, causing the PVs to swell as they accumulate undigested or partially digested materials. This accumulation, coupled with membrane destabilization, compromises the normal digestive functions of the parasite.

It is well-documented that various drugs, including aminoglycoside antibiotics, can affect lysosomal permeability through ROS-mediated mechanisms. Studies have shown that ROS production can induce lysosomal membrane permeabilization (LMP) and apoptosis, leading to cellular damage, in different cell lines (He et al., 2023). ROS induce LMP indirectly via the Fenton reaction, which involves iron, and through the activation of phospholipase A2, both of which destabilize the lysosomal membrane. Additionally, ROS generate hydrogen and hydroxide ions, which can directly oxidize the lipid bilayer of the lysosomal membrane, resulting in its rupture (Yu et al., 2003; He et al., 2023). Similarly, the destabilization of the PVs membranes in *Giardia* could lead to leakage and further expansion as the cell attempts to mitigate the damage. These changes could be characteristic of the early apoptosis state.

Alternatively, ROS can disrupt the endosomal-lysosomal pathway by impairing vesicular transport and key fusion proteins. This disruption can hinder the proper maturation of endosomes into lysosomes and disturb the lysosomal fusion-fission cycle, leading to compromised cellular processes (Ravi et al., 2016; Saffi et al., 2021).

To further explore the effects of **P-2** on the survival of *Giardia*, we conducted cell cycle analysis using flow cytometry. Unlike other eukaryotic cells, *G. lamblia* cells are predominantly seen in the G2/M phase in *in vitro* cultures (Poxleitner et al., 2008). It has been well described that synchronized cell populations are needed when studying processes dependent on a specific cell cycle stage. However, many of the drugs used to achieve that synchronization display several adverse side effects on cell structures and functions, many of which remains after drug release (Kim et al., 2023). The cycle assays without any drug pre-treatment were conducted to avoid any pharmacological interactions and reproduce similar treatment conditions among the different experiments. At the assayed concentrations, **P-2** exerted a remarkable arrest in G1/S subpopulations. All treatment conditions significantly increased the subG1 subpopulations following caspase-independent programmed cell death.

Due to the growing number of cases resistant to treatment with MTZ and other chemotherapeutic agents, such as benzimidazoles and pamoate salts, substantial efforts have been directed toward finding alternatives for *Giardia* infection therapy. One approach has been exploring drug combinations to enhance efficacy and shorten treatment duration, a strategy employed against various bacterial, viral, and parasitic infections (Hausen et al., 2011; Vargas Rigo et al., 2019; Murugaiyan et al., 2022). In this study, combining the IC_50_ values of MTZ and **P-2** resulted in a marked increase in the inhibition of trophozoite proliferation. The observed synergistic effect may be attributed to the fact that both **P-2** and MTZ induce apoptosis through ROS production, though they cause distinct ultrastructural damage to the trophozoites. Using MTZ in conjunction with natural extracts like **P-2** offers a promising strategy to enhance giardicidal activity while potentially reducing MTZ side effects through lower dosing.

On the other hand, the activity of this extract against *G. lamblia* GS/M (genotype B) trophozoites underscores the importance of considering the genetic diversity of the parasite in developing effective treatments. Genotypes A and B show significant differences in virulence and infection capabilities, with genotype B associated with greater pathogenicity in animal models and humans (Nash et al., 1987; Bénéré et al., 2012; Cacciò et al., 2018). This finding is particularly relevant given that effective giardiasis treatment may be affected by the variability in drug sensitivity among different strains.

Future research will focus on isolating and identifying the active compounds within the *Attalea butyracea* fruit extract (**P-2**). Understanding the specific phytochemicals responsible for the antiparasitic activity could lead to the development of more targeted therapies. The HPLC fingerprint profile obtained in the current study provides a preliminary identification of the chemical diversity within the extract, highlighting prominent peaks that may correspond to bioactive compounds. Bioassay-guided fractionation, employing techniques such as vacuum liquid chromatography on silica gel, gas chromatography, liquid chromatography, and preparative HPLC, will be necessary to isolate and identify individual bioactive compounds. Additionally, spectroscopic methods like mass spectrometry and nuclear magnetic resonance will aid in finding the molecular structures of the isolated compounds. However, these experiments will require a prolonged timeframe and are beyond the scope of this work.

The safety profile of **P-2** will continue to be evaluated alongside other studies. *Attalea butyracea* has a long history of traditional use in local diets, where it is consumed as a source of healthy fats and proteins, with no reported adverse effects (Cordero et al., 2009; Bernal et al., 2010). This widespread consumption supports its safety for human use. Earlier studies have proved that fruit extracts from other species within the *Attalea* genus, such as *Attalea phalerata* and *Attalea speciosa*, did not show cytotoxicity *in vitro* and *in vivo* (De Lima et al., 2016; Lima et al., 2023; Acácio et al., 2024). Moreover, *Attalea butyracea* extracts have demonstrated chemopreventive potential (Farag et al., 2019) and have shown no cytotoxicity *in vitro* at a maximal concentration of 50 μg/mL in MCF-7, H-460, and SF-268 cell lines (Olmedo et al., 2018), further reinforcing its safety and therapeutic potential. However, despite these promising *in vitro* results, the comprehensive safety of **P-2** needs to be assessed in more complex systems. Therefore, evaluating the extract’s efficacy in animal models of *Giardia* infection will be necessary. *In vivo* studies would provide insights into the extract’s pharmacokinetics, bioavailability, and potential toxicity, helping to find whether it can be translated into an effective treatment for humans. These directions expand the understanding of the antiparasitic potential of *Attalea butyracea* extract and open new avenues for developing innovative treatments for parasitic infections.

## 5 Conclusion

This study investigates the *in vitro* giardicidal activity of 15 crude extracts derived from plants native to the Colombian Amazon, highlighting the potential of these natural resources in combatting *Giardia lamblia* infections. Notably, we present, for the first time, the giardicidal effects of fruit extracts from *Astrocaryum chambira*, *Attalea butyracea*, and *Bactris gasipaes*. Our findings reveal the possible mechanisms of action of the crude extract from *Attalea butyracea* and its synergistic effect when combined with metronidazole. This synergy enhances therapeutic efficacy and offers a promising strategy to address the challenges of drug resistance. Furthermore, the effectiveness of this extract against *Giardia* GS/M strains demonstrates its broad-spectrum potential as a therapeutic agent. Overall, our results underscore the promise of Colombian plant extracts as effective treatments for giardiasis, paving the way for further research and generating novel therapeutic options.

## Data Availability

The original contributions presented in the study are included in the article/Supplementary material, further inquiries can be directed to the corresponding authors.

## Footnotes

^1^http://www.atcc.org, accessed on 1 March 2008.

^2^ http://fiji.sc/wiki/index.php/Fiji

^3^ http://www.graphpad.com
