# Supplementary material for: Antiparasitic activity of Colombian Amazon palm extracts against Giardia lamblia trophozoites: insights into cellular death mechanisms
Source: Front Microbiol. 2025 Mar 19;16:1523880. doi: 10.3389/fmicb.2025.1523880 (PMC11961968; doi:10.3389/fmicb.2025.1523880)
Supplement: Supplementary file 1 [file Table_1.docx]

**Supplementary material**

**Antiparasitic activity of Colombian Amazon palm extracts against *Giardia lamblia* trophozoites: Insights into cellular death mechanisms.**

Juan Javier García-Bustos^1,2,†^, Gabriel Luna Pizarro^3,†^, Rocío G. Patolsky^3^, Mariana Belén Joray^4^, Vivian Villalba-Vizcaino^2^, Paula Galeano^1^, Fabián Espitia-Almeida^5,6^,  Marco Correa Múnera^1^, Mehmet Oztürk^7^, Andrea S. Rópolo^3^, Contanza Feliziani^3^, María Carolina Touz^3,*^ and Jerónimo Laiolo^3,8,*^

^1^Universidad de La Amazonia, Caquetá, Florencia 180002, Colombia.

^2^ Universidad del Magdalena SUE-Caribe, Magdalena, Santa Marta 470004, Colombia.

^3^Instituto de Investigación Médica Mercedes y Martín Ferreyra, Consejo Nacional de Investigaciones Científicas y Técnicas (INIMEC-CONICET), Universidad Nacional de Córdoba, Córdoba 5016, Argentina.

^4^Centro de Investigación y Desarrollo en Inmunología y Enfermedades Infecciosas, Consejo Nacional de Investigaciones Científicas y Técnicas (CIDIE-CONICET-UCC), Universidad Católica de Córdoba, Córdoba X5016DHK, Argentina.

^5^Centro de Investigaciones en Ciencias de la Vida, Facultad de Ciencias Básicas y Biomédicas, Universidad Simón Bolívar, Barranquilla 080005, Colombia.

^6^Facultad de Ciencias Básicas, Programa de Biología, Universidad del Atlántico, Puerto Colombia 081007, Colombia.

^7^Mugla Sitki Koçman University, Mugla, Turquía.

^8^Universidad Católica de Córdoba, Córdoba X5016DHK, Argentina.

^†^ Equal contribution and first authorship.

***Corresponding Author:** Prof. Dr. Jerónimo Laiolo, Instituto de Investigación Médica Mercedes y Martín Ferreyra, INIMEC (CONICET), Universidad Nacional de Córdoba. Friuli 2434, 5000, Córdoba, Argentina. Phone-fax: (54) (351) 468-1466/ 54-351-4681465 and Universidad Católica de Córdoba, Avda. Armada Argentina 3555, X5016DHK, Córdoba, Argentina. *E-mail address:* [jlaiolo@immf.uncor.edu](mailto:jlaiolo@immf.uncor.edu) and [jeronimolaiolo@ucc.edu.ar](mailto:jeronimolaiolo@ucc.edu.ar)

* **Corresponding Author:** Prof. Dr. María Carolina Touz. *E-mail address:* [ctouz@immf.uncor.edu](mailto:ctouz@immf.uncor.edu)

**Table S1:** Plants from the northern region of the Amazon River basin in Colombia from which extracts were obtained for the evaluation of giardicidal activity.

| **PLANT SPECIES** | **SCIENTIFIC NAME** | **FAMILY** | **VERNACULAR NAME** | **YIELD (%)** | **STATUS**^a^ | **VOUCHER: HUAZ NUMBER** |
| --- | --- | --- | --- | --- | --- | --- |
| *Astrocaryum chambira* | *Astrocaryum chambira* Burret, 1934 | Arecaceae | Coco cumaré | 2.7% | N | 23373 |
| *Attalea butyracea* | *Attofea butyracea* (Mutis ex L. f.) Wess. Boer, 1988 | Arecaceae | Canambo | 7.1% | N | 23410 |
| *Bactris gasipaes* | *Bactris gasipaes* Kunth, 1815 | Arecaceae | Chontaduro | 3.0% | N | 23361 |
| *Cecropia ficifolia* | *Cecropia ficifolia* Warb. ex Snethl., 1923 | Urticaceae | Yarumo | 23.8% | N | 23374 |
| *Inga edulis* | *Inga edulis* Mart., 1837 | Fabaceae | Guamo | 14.6% | N | 23414 |
| *Mangifera indica* | *Mangifera indica* L., 1753 | Anacardiaceae | Mango | 7.2% | I | 23365 |
| *Manihot esculenta* | *Manihot esculenta* Crantz, 1766 | Euphorbiaceae | Yuca dulce | 0.9% | N | 23366 |
| *Mauritia flexuosa* | *Mauritia flexuosa* L.f., 1782 | Arecaceae | Canangucha | 2.6% | N | 25001 |
| *Musa* sp. | *Musa* L.,1753 | Musaceae | Banano | 2,1% | Cult. | 20201 |
| *Psidium guajava* | *Psidium guajava* L., 1753 | Myrtaceae | Guayaba | 10.5% | Cult | 23388 |
| *Renealmia alpinia* | *Renealmia alpinia (Rottb.)* Maas, 1975 | Zingiberaceae | Achira de monte | 3.5% | N | 23378 |
| *Zygia longifolia* | *Zygia longifolia* (Humb. & Bonpl. ex Willd.) Britton & Rose, 1928 | Fabaceae | Carbón | 5.2% | N | 23395 |

^a^Cult: cultivated; I: introduced; N: native

**Table S2:** HPLC peak data for the hydroethanolic *Attalea butyracea* fruit extract.

**
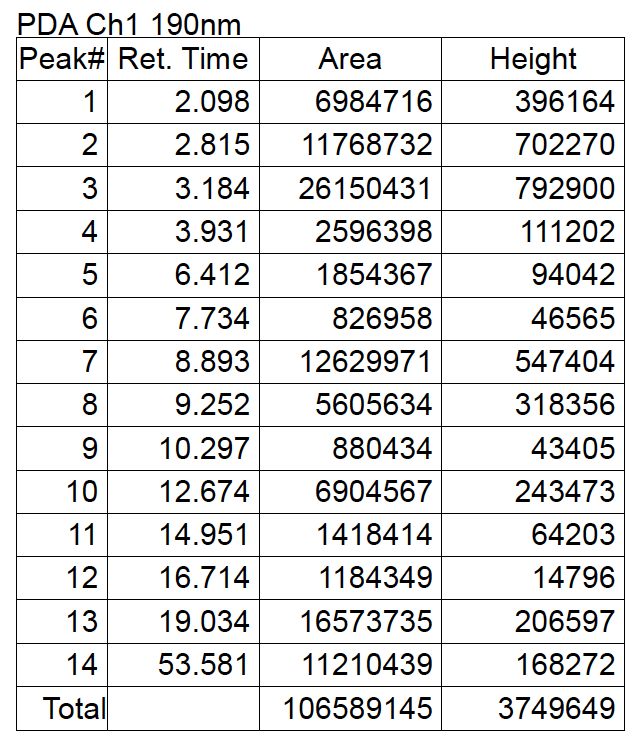
**
